# Commentary: Pandemic exposure, post-traumatic stress disorder, conflict behaviors, and online aggressive behaviors among college students during the COVID-19 pandemic: Examining the moderating role of gender

**DOI:** 10.3389/fpsyt.2022.1006330

**Published:** 2022-09-12

**Authors:** Samira Feizi, Frank Elgar, Michelle Lonergan, Kayla Eisenberg, Nesrine Rahmouni, Alain Brunet

**Affiliations:** ^1^Department of Psychology, McGill University, Montreal, QC, Canada; ^2^Faculty of Social Sciences, School of Psychology, University of Ottawa, Ottawa, ON, Canada; ^3^Department of Psychiatry, McGill University, Montreal, QC, Canada; ^4^Translational Neuroimaging Laboratory, McGill University Research Centre for Studies in Aging, Montreal, QC, Canada; ^5^Research Centre of the Douglas Mental Health University Institute, Montreal, QC, Canada

**Keywords:** PTSD, COVID-19, PCL-5, pandemic, college student

We read with interest the recent study by Zhen et al. (1), titled “Pandemic Exposure, Post-traumatic Stress Disorder, Conflict Behaviors, and Online Aggressive Behaviors Among College Students During the COVID-19 Pandemic: Examining the Moderating Role of Gender.” Their online survey of 1,153 college students found that *pandemic exposure* relates to *conflict behaviors* and *online aggressive behaviors* through posttraumatic stress disorder (PTSD) symptoms. The authors' efforts to contribute to a growing body of knowledge about the psychological consequences of the *pandemic* are laudable. However, conceptual and measurement issues cast uncertainty on their main findings. This letter describes some of our concerns.

PTSD is defined as a “pathological response to the experience of a life-threatening event” [(2); p. 97]. Given this definition, the measure of pandemic exposure used in this research may not have adequately measured “trauma” exposure given that the items did not necessarily capture the “life-threatening” criterion. The weak correlations found between pandemic exposure and symptoms (*r*s = −0.08 to 0.05) suggest that any PTSD in this sample of college students was unrelated to COVID-19. In fact, it appears that participants in this study had mean PTSD scores comparable to those of military veterans (3); but it is unclear what “trauma” was experienced. This is important because when participants in a recent study were asked whether they had experienced trauma as a consequence of COVID-19, only 7% responded affirmatively (4). During the COVID-19 pandemic, it was mostly front-line health care workers and individuals who witnessed a death or had a near-death experience due to COVID-19 who were at risk of developing PTSD (5). Therefore, the theoretical model presented by the authors is inconsistent with the known epidemiology of PTSD (6). A causal model of PTSD would naturally describe its symptoms as the outcome, not as mediators on a causal chain to something else.

In addition to this conceptual issue, certain methodological aspects led us to question the robustness of the findings. The authors measured symptoms using the well-known PTSD Checklist (i.e., PCL-5). There are two concerns with how this measure was used by the authors that challenge its construct validity. First, the PCL-5 is a 20-item self-report measure of PTSD symptom severity in the past month, and items are rated on a 5-point Likert scale ranging from 0 (*not at all*) to four [*extremely*; (7)]. However, the authors asked participants about symptoms in the preceding 2 weeks with the modified range of 0 (*not at all/only once*) to four (*almost every day*) on each item. It is possible their unvalidated version of the PCL-5 measured some aspects of PTSD but did not correspond to DSM-5 criteria.

The second concern involves PTSD inclusion criteria. One way to calculate PTSD severity is by the sum of items on the PCL-5. However, the PCL-5 can also be used to obtain a provisional PTSD diagnosis according to DSM-5 criteria, which requires individuals to endorse at least one intrusive symptom (questions 1–5), one avoidance symptom (questions 6–7), two arousal cognition and mood symptoms (questions 8–14), and two arousal and reactivity symptoms (questions 15–20; 7). It is unclear how many participants in the study met these criteria for a possible PTSD diagnosis, given how the measure was used. Arguably, given these fundamental problems regarding PTSD measurement, the construct validity of the questionnaire is reduced.

Finally, although *pandemic exposure* positively predicted PTSD symptoms and is statistically significant, the interpretation of the results requires careful consideration. A closer look at the associations between these variables shows that *pandemic exposure* is only modestly associated with PTSD symptoms. Even though there are substantially significant associations between *pandemic exposure* and PTSD symptoms, namely intrusive (*b* = 0.07), NACM (*b* = 0.05), and hyperarousal symptoms (*b* = 0.06), the beta coefficients are very small. Approximately 95% of the variance of PTSD symptoms was not explained by *pandemic exposure*. Furthermore, the magnitude of the associations between PTSD symptoms and *conflict* and *online aggressive behaviors* was also very small (8). Given the large sample size (*N* = 1,153), we believe these small associations between study variables may not reflect the true relationship but rather an excess of statistical power.

These problems cast doubt on the validity of the study's findings. But other points of confusion remain. For instance, since everyone has experienced the COVID-19 pandemic, what is the precise definition of *pandemic exposure*? Why did the authors use a *conflict behaviors* scale with Chinese college students that was previously found to be unreliable in this population (9)? Why were moderating effects of gender not tested directly? A visual comparison of results from males and females in gender-stratified analyses does not determine whether statistical interactions with gender uniquely predicted symptoms, conflict, or aggression. What is the meaning of a statistical test of model fit in which the degrees of freedom is zero, and the confirmatory fit index is a perfect 1.00? Finally, the authors indicate that data from the questionnaires was collected using choice-forced methods. However, does this mean that participants were forced to respond to each item (i.e., forced-choice), or that each questionnaire had a designated response set from which to choose from (i.e., choice-forced)? The terminology used by the authors is unclear, and if their data collection method consisted of a forced-choice approach, the implications of this for social desirability bias should be addressed.

In conclusion, this study tackled an important topic and an existing research gap that could direct public health initiatives during a public health emergency. Unfortunately, considering theoretical and methodological reservations that put doubt on the study's results and conclusions, the authors failed to deliver on this objective and instead added noise to small but important literature on the psychological consequences of the COVID-19 pandemic. We encourage the authors to address these issues with a revised methodological model and clarify the ambiguities observed in the research.

## Author contributions

SF: writing–original draft and conceptualization. FE: writing–review and editing. AB and ML: conceptualization–review and editing. KE and NR: review. All authors contributed to the article and approved the submitted version.

## Conflict of interest

The authors declare that the research was conducted in the absence of any commercial or financial relationships that could be construed as a potential conflict of interest.

## Publisher's note

All claims expressed in this article are solely those of the authors and do not necessarily represent those of their affiliated organizations, or those of the publisher, the editors and the reviewers. Any product that may be evaluated in this article, or claim that may be made by its manufacturer, is not guaranteed or endorsed by the publisher.
